# A multi-laboratory study of diverse RSV neutralization assays indicates feasibility for harmonization with an international standard

**DOI:** 10.1016/j.vaccine.2017.04.053

**Published:** 2017-05-25

**Authors:** Nancy Hosken, Brian Plikaytis, Carrie Trujillo, Kutub Mahmood, Deborah Higgins, the Participating Laboratories Working Group

**Affiliations:** a PATH, Seattle, WA, United States; b BioStat Consulting LLC, Jasper, GA, United States

**Keywords:** CT, Clinical trial, NI, Natural infection, IS, International standard, Respiratory syncytial virus, Antibodies, Neutralization, International standard, Vaccine, Assay

## Abstract

A current barrier to the standardized evaluation of respiratory syncytial virus (RSV) vaccine candidates is the wide variety of virus neutralization assay formats currently in use for assessing immunogenicity. Assay formats vary widely in labor intensiveness, duration, and sample throughput. Furthermore, the cell lines and virus strains used are not consistent among formats. The purpose of this multi-laboratory study was to assess the variability across a diverse array of assay formats that quantitate RSV neutralizing antibodies. Using a common specimen panel, the degree of overall agreement among existing assays was evaluated to inform on the need for harmonization of assay results. A total of 12 laboratories participated in the blinded survey study by testing a panel comprised of 57 samples chosen to span the reportable titer range of the assays. An independent statistical analysis was conducted to measure overall agreement of assay results. This analysis showed that precision was consistently high, whereas agreement varied widely among assays. To examine whether agreement could be improved, we conducted a harmonization exercise using a variety of sample types as pseudo standards. The results showed that the level of agreement could be improved, and provided information on the suitability of samples for developing an international standard.

## Highlights

• A wide variety of RSV neutralization assay formats are currently used by the field.
• This study assessed the current level of agreement between twelve assay formats.
• Results showed precision was consistently high, whereas agreement varied widely.
• Results indicated harmonization could be improved with an international standard.
• Results provided information on samples that may be appropriate for IS development.

## 1 Introduction

Respiratory syncytial virus (RSV) is a leading cause of lower respiratory tract infection globally and results in significant morbidity and mortality among infants, children, elderly, and immunocompromised individuals [1], [2]. Young children are especially susceptible to RSV infection, with 60–70% acquiring RSV within their first year of life [3], and 98% by two years of age [4], [5]. In 2010, RSV infection resulted in an estimated 33.8 million new episodes of acute lower respiratory illness in children less than five years old, 3.4 million hospital admissions, and 253,000 deaths—99% of which occurred in low-resource countries [6], [7].

Treatment for RSV respiratory illness is provided through supportive care, including clearing of nasal pathways, hydration, and oxygen therapy in advanced cases [8]. In pediatric patients at risk of developing RSV disease, immunoprophylaxis with RSV neutralizing antibodies (nAb) (*viz*. RSV-IVIG, palivizumab, and motavizumab) have been shown to provide protection against serious RSV illness [9], [10]. These antibody-based interventions represent proof-of-concept that vaccines capable of inducing RSV neutralizing antibodies could confer protection against severe disease. The availability of a low-cost prophylactic intervention has the potential to dramatically reduce the global burden of RSV. Several promising RSV interventions are currently in clinical development [11], including two candidates in Phase 3 clinical trials initiated in 2015 [12], [13].

For RSV vaccines targeting induction of humoral immunity, assays evaluating antibody function will be used to identify correlates of protection. RSV-specific functional antibodies measurement is a critical metric and neutralization assays are the standard methodology employed for this measurement [14], [15]. Multiple assay formats for quantitating RSV nAb have been developed over time as alternatives to the labor intensive, lengthy, and relatively low-throughput classic plaque reduction neutralization test (PRNT) [16]. These assay formats vary in terms of the time allowed for virus-cell interactions. Longer interaction timeframes (multiple days) evaluate multiple rounds of virus replication, whereas shorter interaction timeframes (≤ 1 day) may only assess a single round of virus replication. Assay formats also vary widely by other parameters, such as viral strains, cell lines, use of complement, and results reporting, leading to potential variation in the nAb titers generated; therefore making comparing results across vaccine candidates difficult.

Currently, with more than 15 RSV vaccine candidates at various stages of clinical development [17], assay harmonization to allow comparison of results across assay formats is urgently needed. To inform next steps, PATH conducted a multi-laboratory survey study of diverse RSV neutralization assay formats using a common specimen panel to assess the degree of agreement and feasibility for harmonization. Laboratories from industry, academia, and regulatory agencies participated in this study. Study results indicated consistently high precision, but variable agreement, of nAb titers between laboratories. Further analyses demonstrated feasibility for a standard to improve agreement and harmonize nAb titers across assay formats, and also provided information on sample types that may be appropriate for development of an international standard (IS).

## 2 Materials and methods

### 2.1 Study design

Twelve laboratories participated in this study, each using their existing RSV neutralization assay method (Supplementary Table 1). For anonymity, the blinded alphabetic identifications A through L were randomly assigned to these laboratories and do not correspond to the laboratories’ randomly assigned blinded numeric identifications 1 through 12 in Supplementary Table 1.

Each participating laboratory received a blinded and randomized specimen panel (described in Section 2.2), performed its existing RSV neutralization assay per its standard procedure, and treated the specimen panel as ‘unknown’ samples. No alteration in the preparation of replicates (if done), analysis of data, nor reporting of the results was requested. Each laboratory reported a single valid nAb titer for each sample to PATH using a standardized data collection form. Each laboratory was kept blinded to the results reported by all of the other laboratories.

### 2.2 Specimen panel

**Fig. 1:**
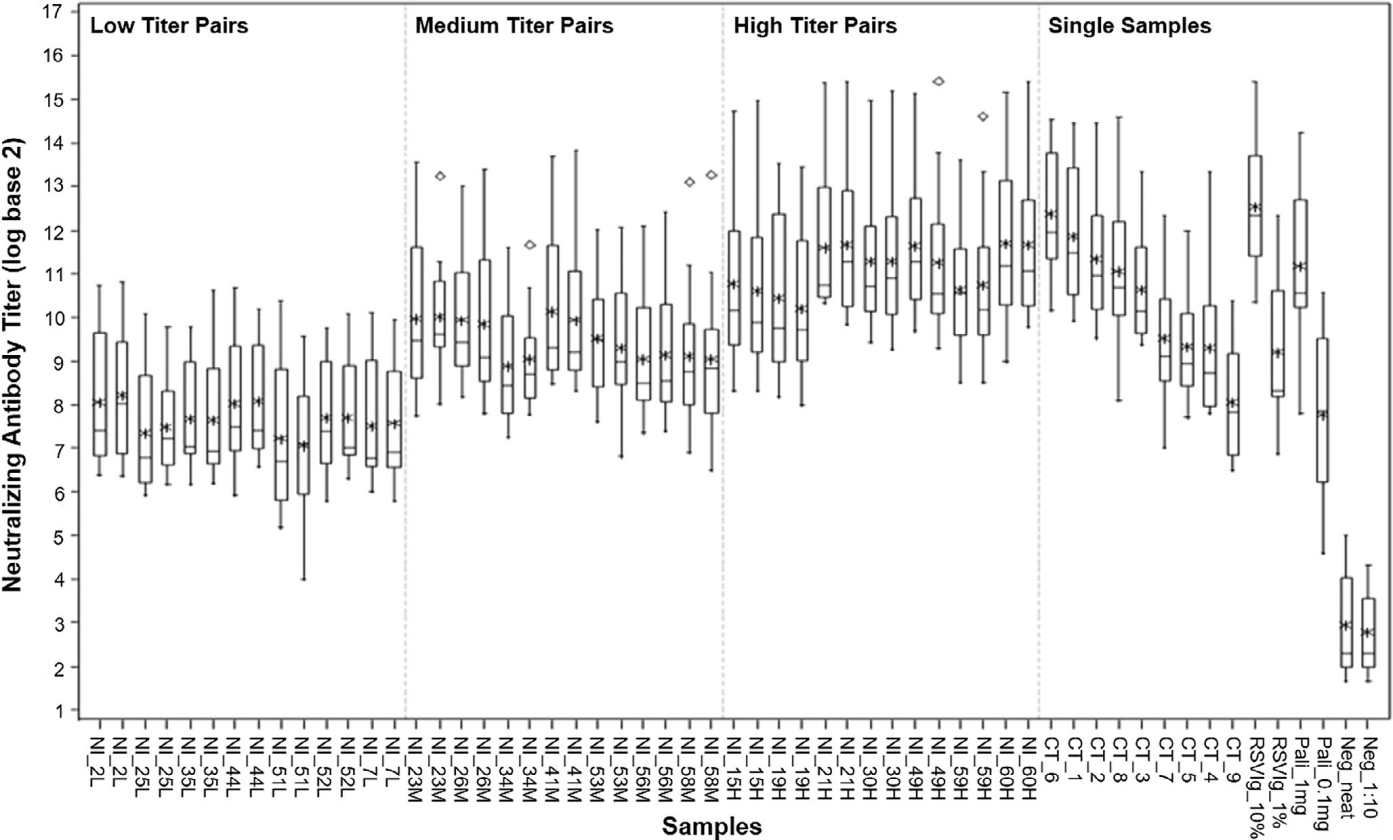
Box plots showing the distribution of neutralizing antibody titers measured for each sample by the laboratories. The box plots display the degree of agreement for each of the 57 samples in the specimen panel. In these box plots, the box is defined by the interquartile range (IQR) – the 25th and 75th percentiles of the distribution; the horizontal line within the box represents the median (50th percentile); and the asterisk represents the mean. Vertical lines extend to the most extreme observation that is less than 1.5 × the IQR, and the open diamonds correspond to moderate outliers (between 1.5–3.0 × IQR). There are no severe outliers (>3.0 × IQR; open squares). The 21 replicated natural infection (NI) serum samples are divided into low, medium, and high titer groups of 7 samples per group on the left-hand side of each plot. The 15 single (non-replicated) samples are grouped on the right hand side of the box plots figure.

The specimen panel consisted of 57 blinded and randomized samples. Individual sera from 60 normal healthy adults presumed to have a history of natural infection (NI) with RSV were obtained from US Food and Drug Administration (US FDA)-licensed blood centers with appropriate informed consent. These samples were randomly assigned the blinded numerical identifiers 1 through 60, and are hereafter referred to as NI sera. Two laboratories screened the 60 NI sera using their RSV neutralization assays to assess their nAb titers. Twenty-one of the NI sera that had titers spanning the range of these RSV nAb assays were selected for inclusion in the specimen panel. These 21 samples were divided into low, medium and high titer groups of 7 samples per group (Fig. 1) and were anonymously duplicated within the specimen panel.

A purified RSV immune globulin (Ig) prepared from pooled plasma collected from healthy donors, some of which had likely been naturally infected with RSV, was provided by US FDA. This material is available at no cost to the research community through BEI Resources [18]. The material was included at 1X (10% Ig) and 0.1X (1% Ig) concentrations to serve as positive controls, which were respectively assigned the identifiers RSVIg_10% and RSVIg_1%. US FDA also provided human immunoglobulin-depleted serum (Sunnylab, Sittingbourne, United Kingdom; Cat. SF142-2). This material was used at 1X and 0.1X concentrations as negative controls, which were respectively assigned the identifiers Neg_neat and Neg_1:10. The 0.1X concentrated sample of each material was prepared by diluting the 1X concentrated sample 10-fold with sterile phosphate-buffered saline.

Medimmune (Gaithersburg, Maryland) contributed palivizumab, an RSV fusion (F) protein site II-specific neutralizing monoclonal antibody [19]. A 10 mg/mL solution of palivizumab was prepared using sterile deionized water for the reconstitution of the lyophilized stock material. Then 1.0 mg/mL and 0.1 mg/mL palivizumab samples were prepared by serial dilution (1:10) using sterile deionized water as diluent. These two samples were assigned the identifiers Pali_1mg and Pali_0.1mg.

An RSV vaccine developer contributed nine pooled human serum samples, each comprised of equal volumes of five individual samples, from a clinical trial (CT) of an investigational RSV vaccine. Of the nine pooled samples, six were prepared from sera samples from subjects who received a single dose of an RSV F protein-containing candidate vaccine (individual samples were collected between 30 and 180 days post vaccination) and three were prepared from samples taken after administration of a placebo. All samples were collected with appropriate informed consent. These pooled CT samples were randomly assigned the blinded numerical identifiers 1 through 9.

The specimen panels were prepared by Lampire Biological Laboratories (Everett, Pennsylvania) per PATH’s detailed instructions. Prior to dispensing into vials (250 µL/vial) and labeling, the 57 samples in the specimen panel were randomized and assigned the blinded numerical identifiers 1 through 57. Lampire shipped a frozen (≤−60°C) specimen panel to each participating laboratory. After completing sample testing, the laboratories returned their remaining portions of the specimen panels to Lampire for destruction prior to being informed of final study results.

### 2.3 Statistical analysis

An independent statistical analysis of the dataset was performed. The final dataset for analysis consisted of a single nAb titer value for each of the 57 samples in the specimen panel from each of the 12 laboratories. All titer values were transformed to log, base 2, (log_2_) values prior to analysis. A single pair of duplicate samples from one laboratory exhibited an excessively high within-sample coefficient of variation (CV) value when compared to samples assayed by the other laboratories. Further examination of this particular pair revealed one of the observations to be an extreme outlier, and it was excluded from the final dataset prior to analysis.

Box plots were employed to display the distribution of assay results for each sample, aggregated over all of the laboratories. Scatter plots were employed to display the degree of correlation and agreement between laboratories. The level of agreement between pairs of laboratories was measured using the accuracy coefficient (*C_a_*) due to Lawrence Lin [20]. Precision (a measure of how far a set of observations deviates from a straight line) was measured using Pearson’s correlation coefficient (*r*) for each comparison. Lin’s concordance correlation coefficient (*r_c_*), the product of *r* and *C_a_*, was employed to measure the overall level of agreement between laboratories. Titer values for the 21 duplicated NI serum samples in the panel were used to measure within-sample and within-laboratory variances (see Supplementary Figs. 1 and 2 for diagrams of the approaches used for this analysis). A mixed-model analysis of variance (ANOVA) was used to quantify these variances, expressed as CV values. Sample, laboratory, and their interaction were modeled as random effects in this analysis.

A normalization exercise was also performed to examine whether titer harmonization across assay formats could be achieved using a standard. Since a reference standard has not yet been established, the normalization process was simulated using a sample from the panel to serve as the internal standard (hereafter referred to as pseudo internal standard.) The following different sample types were selected to serve as pseudo internal standards: (1) NI sera; (2) purified RSV Ig; (3) palivizumab; and (4) CT sera. The titer value for each unknown sample was normalized using the following simple formula: Titer × (‘Defined value for internal standard’/‘Laboratory measured value for internal standard’). ‘Titer’ was the titer value measured by the laboratory for the unknown sample. ‘Laboratory measured value for internal standard’ was the titer value measured by the laboratory for the sample used as the pseudo internal standard. ‘Defined value for internal standard’ was estimated by taking the median value of titer results for the sample used as pseudo internal standard assayed by all 12 laboratories. Scatter plot matrices of pre- and post-normalization antibody titer data were produced and values for the statistical coefficients *r*, *C_a_*, and *r_c_* were calculated to assess the degree of agreement between assays. Box plots displaying the distribution of post-normalization titer values for each sample were also produced.

## 3 Results

### 3.1 Agreement analysis results

To assess the degree of variability, box plots were employed to display the distribution of assay results for each sample, aggregated over all of the laboratories (Fig. 1). As expected, the RSV nAb titers for the two Ig-depleted negative control samples (Neg_neat and Neg_1:10) had comparably low mean nAb titers that were substantially below the mean titers for all other samples in the panel. Also as expected, the nAb titers for the two purified RSV Ig (RSVIg_10% and RSV_1%) and two palivizumab (Pali_1mg and Pali_0.1mg) positive control samples were quantifiable in all assays in this study, with approximately 10-fold lower titers for samples having 10-fold lower antibody concentration. In general, the members of each pair of duplicate samples displayed similarities in means, medians, and inter-quartile ranges (IQR) of RSV nAb titer values. A few moderate outliers were identified and are indicated with open diamond symbols on the box plots. They lie between 1.5 and 3.0 × IQR for the following samples: NI_23M (second replicate), NI_34M (second replicate), NI_58M (both replicates), NI_49H (second replicate), and NI_59H (second replicate). These moderate outliers influenced the variance analysis described in Section 3.2.

### 3.2 Variance analysis results

**Fig. 2:**
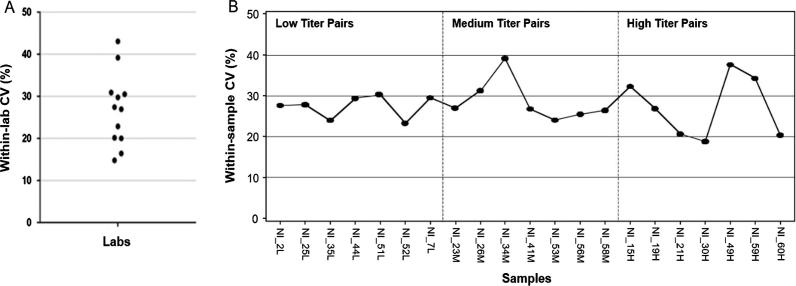
Within-laboratory and within-sample coefficients of variation. A: Within-lab coefficients of variation. In this graph, the closed circles correspond to the within-laboratory percentage coefficients of variation (CV) values for each of the 12 participating laboratories (one circle per lab). B: Within-sample coefficients of variation. In this graph, the closed circles correspond to the within-sample coefficients of variation (CV) values for the 21 duplicated samples in the specimen panel (one circle per duplicated sample).

To assess the degree of variability within each laboratory, the CV was calculated using the RSV nAb titer data for the 21 duplicated NI sera samples in the specimen panel (Fig. 2a). This analysis showed that within-laboratory CV values were generally ≤40% and many were ≤30%.

To assess the degree of within-sample variability, the CV was calculated using the RSV nAb titer data for the 21 duplicated NI sera samples (Fig. 2b). This analysis showed that within-sample CV values ranged from 19% to 39%. No apparent correlation was seen between the observed CV values and antibody titer (low, medium, or high) for the samples.

### 3.3 Normalization analysis results

**Fig. 3:**
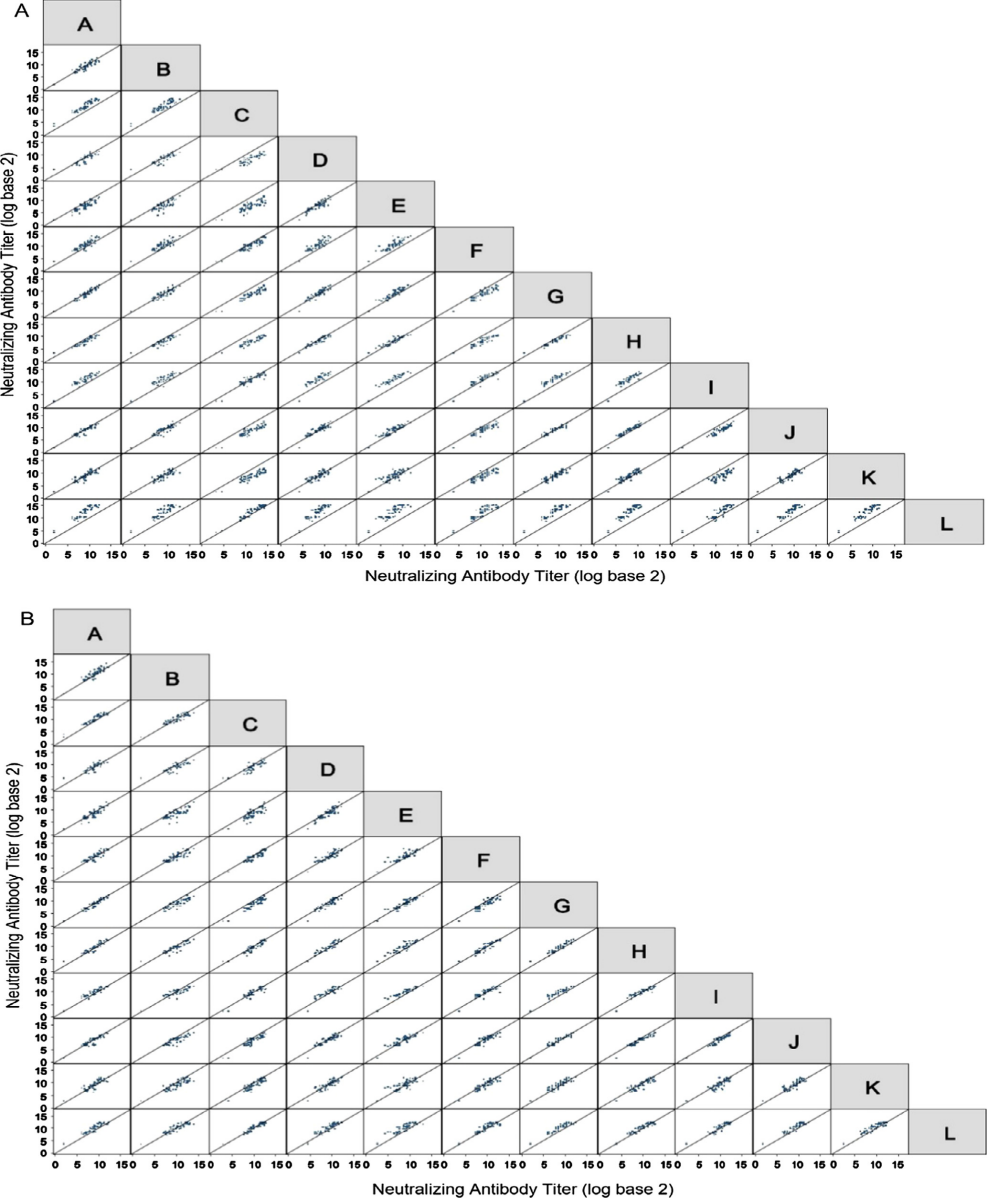
Scatter plot matrices illustrating the degree of agreement between labs before and after normalization. The scatter plot matrices display pairwise comparisons of RSV neutralizing antibody (nAb) titer values from the laboratories (A) before normalization and (B) after normalization using the purified RSV immune globulin (Ig) sample (RSVIg_10%) as the pseudo internal standard. In each scatter plot, each black dot plots the pairwise comparison of the log, base 2, antibody titers for a sample. The solid diagonal line indicates perfect agreement (intercept 0 and slope 1). The laboratories are designated by the blinded alphabetic identifications A-L.

To assess the level of agreement and precision between laboratories, a correlation analysis was conducted by generating a scatter plot matrix of assay titer results. Fig. 3a is a scatter plot matrix showing the relationship of log_2_ RSV nAb titers between each pair of laboratories (A-L) prior to normalization. The diagonal line in each scatter plot is the line of identity (intercept of 0 and slope of 1). The values of three different coefficients (*r*, *Ca*, and *r_c_*) were calculated for each comparison.

The analysis showed that the overall level of agreement between laboratories varied broadly (Fig. 3a and Supplementary Table 2). Twenty-eight of the 66 comparisons had *r_c_* values ≥ 0.800. Precision was consistently high (*r* ≥ 0.800 for all comparisons), whereas the degree of agreement between the different assay formats varied widely (*C_a_* ranged from 0.307 to 0.997).

**Fig. 4:**
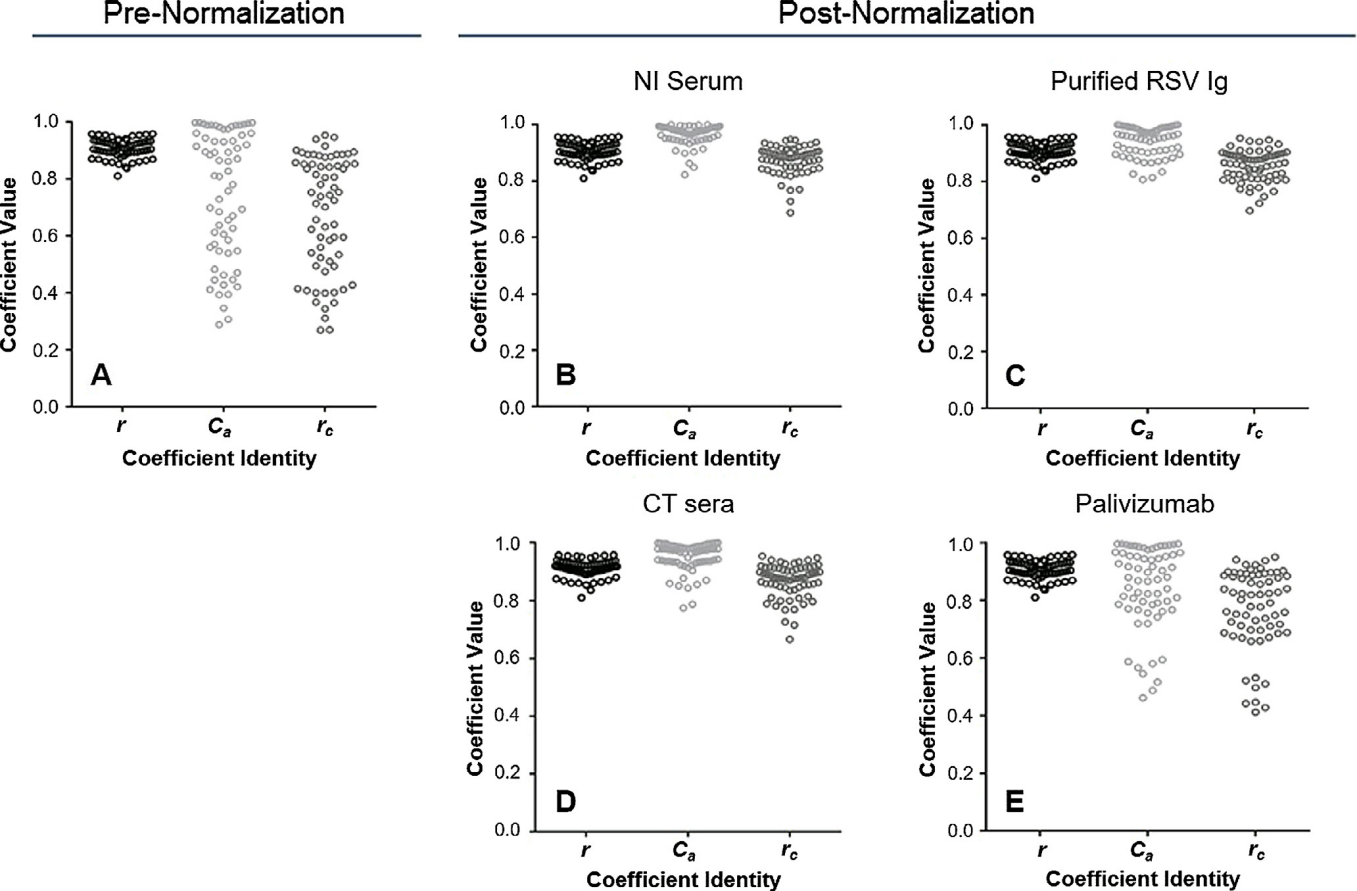
Graphs of statistical coefficient values showing the degree of agreement pre- and post-normalization. In each graph, the values of the statistical coefficients used to quantify precision (*r*), agreement (C_a_), and overall agreement (*r_c_*) are plotted in aggregate. Each open circle plots the value of the indicated statistical coefficient (*r*, *C_a_*, or *r_c_*) for the comparison of antibody titer results for all samples from a pair of laboratories. The values of these statistical coefficients prior to normalization are plotted in graph A. The values of these statistical coefficients after normalization using natural infection (NI) serum (sample NI_56M), purified RSV immune globulin (Ig) (sample RSVIg_10%), clinical trial sera (sample CT_5), and palivizumab (sample Pali_1mg) as pseudo internal standards for normalization are plotted in graphs B, C, D, and E, respectively.

To evaluate the potential for an IS to enable harmonization across assays, a normalization exercise was conducted. To achieve this, each laboratory’s assay results were further analyzed by normalizing the antibody titer values using each of the following sample types: (1) NI serum (individual); (2) purified RSV Ig (pooled); (3) palivizumab; and (4) CT sera (pooled) as a pseudo internal standard. This analysis showed that normalization with any of the four sample types used as pseudo internal standards substantially improved agreement across diverse nAb assay formats and also reduced within-sample variability. As a representative example, Fig. 3b shows the post-normalization scatter plot matrix for the analysis where the RSV Ig_10% sample was used as a pseudo internal standard. Improvements in agreement can be seen with the general movement of the point clouds to center over the diagonal line of identity. Box plots displaying the degree of variability for each sample after normalization using each of the four sample types as a pseudo-internal standard are provided in Supplementary Fig. 3. Improvements in agreement can be seen with the decreased size of the boxes for the samples relative to their sizes in Fig. 1. The greatest improvement in agreement was observed when either a NI serum sample (NI_56M), a CT serum sample (CT_2), or a purified RSV Ig sample (RSVIg_10%) was used as a pseudo internal standard. This effect is illustrated by the plots of the correlation coefficient values for *r*, *C_a_*, and *r_c_* (Fig. 4), in which substantial migration towards perfect agreement (*C_a_* = *1.0*) and concordance (*r_c_* = *1.0*) were observed after normalization. The Pearson correlation coefficient (*r*) values did not change after normalization since the pseudo internal standard was constant for all nAb titer values within a laboratory. Ideally, an internal standard run on each plate would affect the calculations for *r* values; however, running a standard on every plate may not be practical for all assay formats. The degree of harmonization observed when an NI serum sample (NI_56M) was used as the pseudo internal standard was generalizable because we observed comparable improvement in the degree of agreement when many of the other NI serum samples were used as pseudo internal standards (Supplementary Fig. 4). Whether or not the ability of an individual CT sera sample (CT_2) to harmonize results was generalizable was unclear because only nine CT sera samples were available for comparison and because we observed substantial variability in the degree of agreement improvement when each of these CT sera samples was used as a pseudo internal standard (data not shown). Furthermore, the NI sera came from individual donors, while the CT sera were pools created from multiple donors. It is possible that pooling NI sera to create sufficient volume for a standard may increase variability related to its ability to harmonize.

## 4 Discussion

In this multi-laboratory study we surveyed 12 diverse RSV neutralization assay formats using a common specimen panel to assess the degree of agreement and the feasibility for assay output harmonization using a reference standard. To our knowledge, this was the broadest survey study across RSV neutralization assay formats performed to date. Results of this study demonstrated that the degree of precision was consistently high; whereas the degree of agreement varied widely between assay formats. To see that 28 of 66 comparisons had a relatively high level of overall agreement (*r_c_* *≥* 0.8) was encouraging. The results additionally showed that within-laboratory and within-sample CVs for the 21 duplicated NI sera samples fell within an acceptable range (≤40%) for assays having substantial biological complexity. Within-sample CV values ranged from 19% to 39% and the CV values were comparable across the low, medium, and high nAb titer ranges. This observation suggested that NI serum samples having comparably low CV values in multiple assay formats may be identified, which may inform the development of a suitable IS.

To assess the feasibility of improving agreement across assay formats using a standard, a normalization approach was employed using a variety of sample types as pseudo internal standards. Although our normalization approach was imperfect because it did not correct for plate-to-plate variation, the substantial improvement in agreement with certain sample types used as pseudo internal standards strongly suggested that establishing an IS could improve agreement across diverse assay formats and provide information on sample types that may be suitable for developing an IS. The greatest and most consistent improvement was observed when either RSV Ig (10% concentration), NI sera (56M), or CT sera (CT_2) samples were used as the standard. Many of the 21 NI serum samples substantially improved the degree of agreement when used as pseudo standards (Supplementary Fig. 4), providing additional evidence to suggest that NI sera may be suitable for developing an IS. NI serum samples (including the remaining volumes of the 60 NI serum samples from this study) are readily available, suggesting that they may be a pragmatic sample type for developing an IS.

Taken together, our observations strongly suggest that establishing an IS could improve agreement across assay formats, and that identifying sample types that may be suitable for developing an IS is possible. Improved harmonization would enable the ability to look across assay results to allow comparison and prioritization across studies and vaccine candidates. Next steps include conducting an international collaborative study led by the UK National Institute of Biological Standards and Control (Potters Bar, United Kingdom) to evaluate candidate IS materials and assess commutability. This study is targeted to start in early 2017, and approval to implement the RSV IS by the World Health Organization’s Expert Committee on Biological Standardization is targeted for late 2017. In the interim, the purified RSV Ig material used in this study as a pseudo standard might be used to harmonize output across RSV neutralization assay formats. Our rationale for this suggestion includes the following: (1) the observation that this material worked well for this purpose in our study; (2) a large quantity is available to the field at no cost from BEI Resources [19]; and (3) the material will be included in the specimen panel for the collaborative study, which will allow it to be calibrated against the IS. Commutability of reference materials should be assessed across a broad array of sample types, since this was not done in our study. Additional RSV antibody and virus materials are available from BEI Resources that may be useful to laboratories conducting RSV neutralization assays.

## Disclaimers

The findings and conclusions contained within are those of the authors and do not necessarily reflect positions or policies of the Bill & Melinda Gates Foundation. The findings and conclusions in this article are those of the authors and do not necessarily represent the official position of the Centers for Disease Control and Prevention.

## Conflict of interest

None.

## Contribution of each author to this work

DH was responsible for overseeing all work for this study, contributing to study design, interpreting results, and preparing this manuscript. CT coordinated most activities for this study, and contributed to preparing this manuscript. NH coordinated analysis of the dataset, advised on data interpretation, and led the preparation of this manuscript. KM advised on study design, procured the samples for the specimen panel, and contributed to preparing this manuscript. BP advised on study design, performed statistical analysis of the study dataset, advised on data interpretation, and contributed to preparing this manuscript. The Participating Laboratories Working Group advised on study design, performed sample testing in their assays, and provided feedback on this manuscript.

## Appendix A Supplementary material

Supplementary Table 1 Assay format details for the RSV neutralization assays assessed in this study.

Supplementary Table 2 Table of statistical coefficient values prior to normalization.

Supplementary Fig. 1 Diagram illustrating how within-laboratory coefficient of variation values were calculated. Each laboratory (Lab 1 to 12) provided titer data for each of the 21 duplicated NI sera samples in the specimen panel. Each blue bar in the diagram represents a titer result for an NI sera sample replicate. As an example, we first calculated within-sample CV values which measured the deviation of each individual titer result for NI sera sample 1 from the mean of the 2 titer values for this same sample (green box). The same was then done for each of the other NI sera samples assayed by Lab 12. These within-sample CV values were then pooled over all of the NI sera samples assayed by Lab 12 (red box) using a mixed-model analysis of variance (ANOVA) with repeated measures to calculate a overall within-lab CV value for the samples for Lab 12. Sample, lab, and their interaction were modeled as random effects in this analysis. This identical method was used to calculate overall within-lab CV values for the samples for each of the other labs. See Fig. 2a for results of this analysis.

Supplementary Fig. 2 Diagram illustrating how within-sample coefficient of variation values were calculated. Each laboratory (Lab 1 to 12) provided titer data for each of the 21 duplicated NI sera samples in the specimen panel. Each blue bar in the diagram represents a titer result for an NI sera sample replicate. As an example, we first calculated within-sample CV values which measured the deviation of each individual titer value for NI sera sample 1 assayed by Lab 2 from the mean of the two titer values for that same sample (green box). This identical method was used to calculate within-sample CV values for each of the other labs. These values were then pooled over all 12 laboratories within that sample (red box) to calculate an overall within-sample CV value for the laboratories for NI sera sample 1 using a mixed model analysis of variance (ANOVA) with repeated measures. Sample, lab, and their interaction were modeled as random effects in this analysis. This identical method was used to calculate the overall within-sample CV variance values for the laboratories for each of the other NI sera samples. See Fig. 2b for results of this analysis.

Supplementary Fig. 3 Box plots illustrating degree of agreement after normalization. The box plots display the degree of agreement on RSV neutralizing antibody (nAb) titer for each sample after normalization using A) purified RSV immune globulin (Ig) (sample RSVIg_10%); B) natural infection (NI) serum (sample NI_56M); C) clinical trial (CT) sera (sample CT_5); and D) palivizumab (sample Pali_1mg) as the pseudo internal standard. In these plots, the box is defined by the interquartile range (IQR)—the 25^th^ and 75^th^ percentiles of the distribution; the horizontal line within the box represents the median (50th percentile); and the asterisk represents the mean. Vertical lines extend to the most extreme observation that is less than 1.5×IQR, and the open diamonds correspond to moderate outliers (between 1.5-3.0×IQR). The 21 replicated NI serum samples are divided into low, medium, and high nAb titer groups of 7 samples per group on the left-hand side of each plot. The 15 single (non-replicated) samples are grouped on the right hand side of each plot.

Supplementary Fig. 4 Graphs of statistical coefficient values pre- and post-normalization using each natural infection sera sample as a pseudo internal standard. In each graph, the values of the statistical coefficients used to quantify precision (*r*), agreement (C_a_), and overall agreement (*r_c_*,) are plotted in aggregate. Each open circle plots the value of the indicated statistical coefficient (*r*, *C_a_*, or *r_c_*) for the comparison of antibody titer results for all samples from a pair of laboratories. The values of these statistical coefficients prior to harmonization are plotted in graph A. The values of these statistical coefficients after normalization using each of the 21 natural infection (NI) serum samples (2L, 7L, 15H, 21H, 23M, 25L, 26M, 30H, 34M, 35L, 41M, 44L, 49H, 51L, 52L, 53M, 56M, 58M, 19H, 59H, and 60H) as a pseudo internal standard for normalization are plotted in graphs B through V. The post-normalization graphs are grouped by the RSV neutralization titer of the NI sera samples used for normalization as follows: low titer (graphs B to H); medium titer (graphs I to O); and high titer (graphs P to V).
