# Supplementary figures and images for: A multi-laboratory study of diverse RSV neutralization assays indicates feasibility for harmonization with an international standard
Source: Vaccine. 2017 May 25;35(23):3082–8. doi: 10.1016/j.vaccine.2017.04.053 (PMC5439532; doi:10.1016/j.vaccine.2017.04.053)

## Slide 1
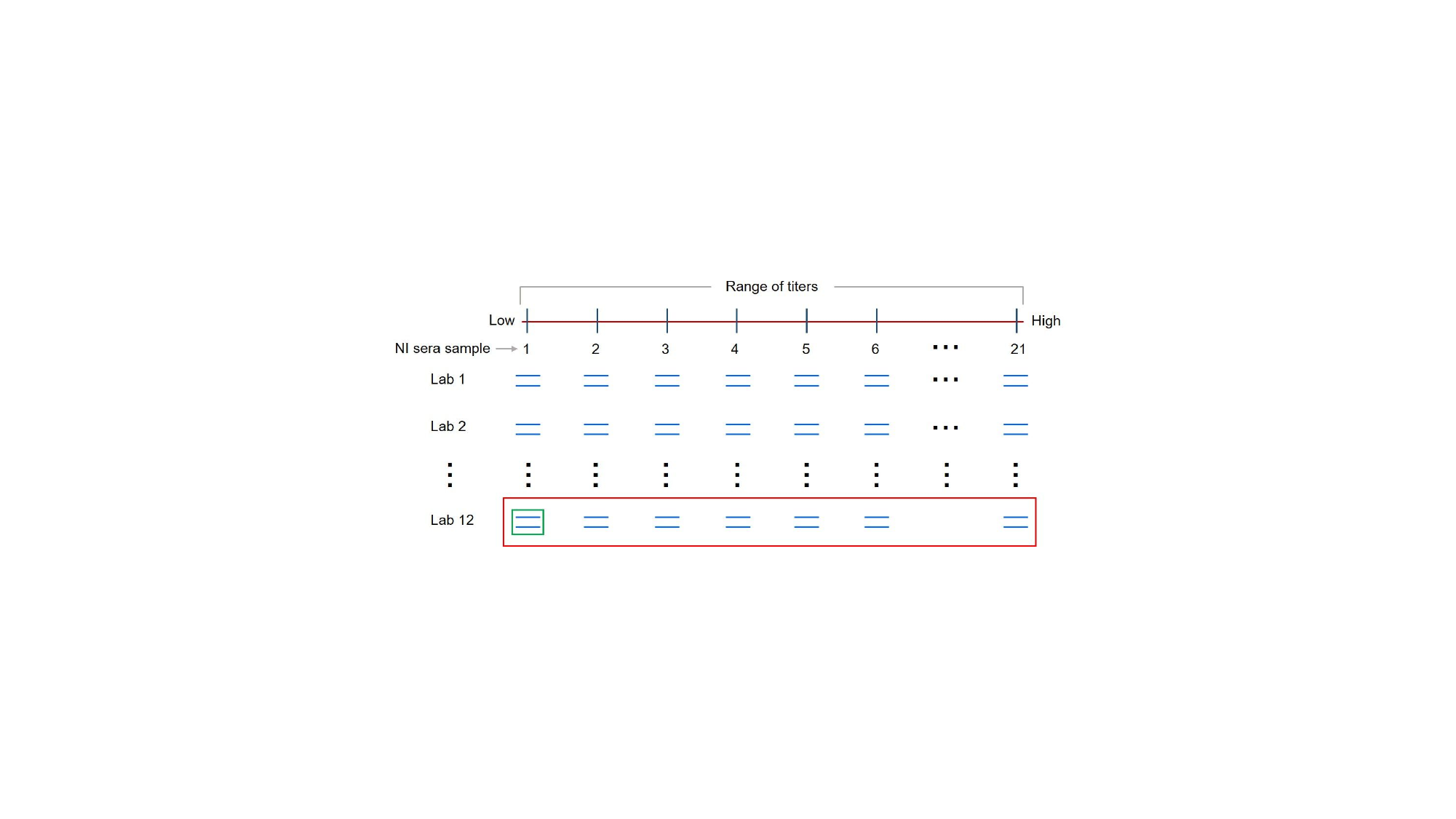

Supplement: Supplementary Fig. 1 — Diagram illustrating how within-laboratory coefficient of variation values were calculated. Each laboratory (Lab 1 to 12) provided titer data for each of the 21 duplicated NI sera samples in the specimen panel. Each blue bar in the diagram represents a titer result for an NI sera sample replicate. As an example, we first calculated within-sample CV values which measured the deviation of each individual titer result for NI sera sample 1 from the mean of the 2 titer values for this same sample (green box). The same was then done for each of the other NI sera samples assayed by Lab 12. These within-sample CV values were then pooled over all of the NI sera samples assayed by Lab 12 (red box) using a mixed-model analysis of variance (ANOVA) with repeated measures to calculate a overall within-lab CV value for the samples for Lab 12. Sample, lab, and their interaction were modeled as random effects in this analysis. This identical method was used to calculate overall within-lab CV values for the samples for each of the other labs. See Fig. 2a for results of this analysis. [file mmc3.pptx]

## Slide 1
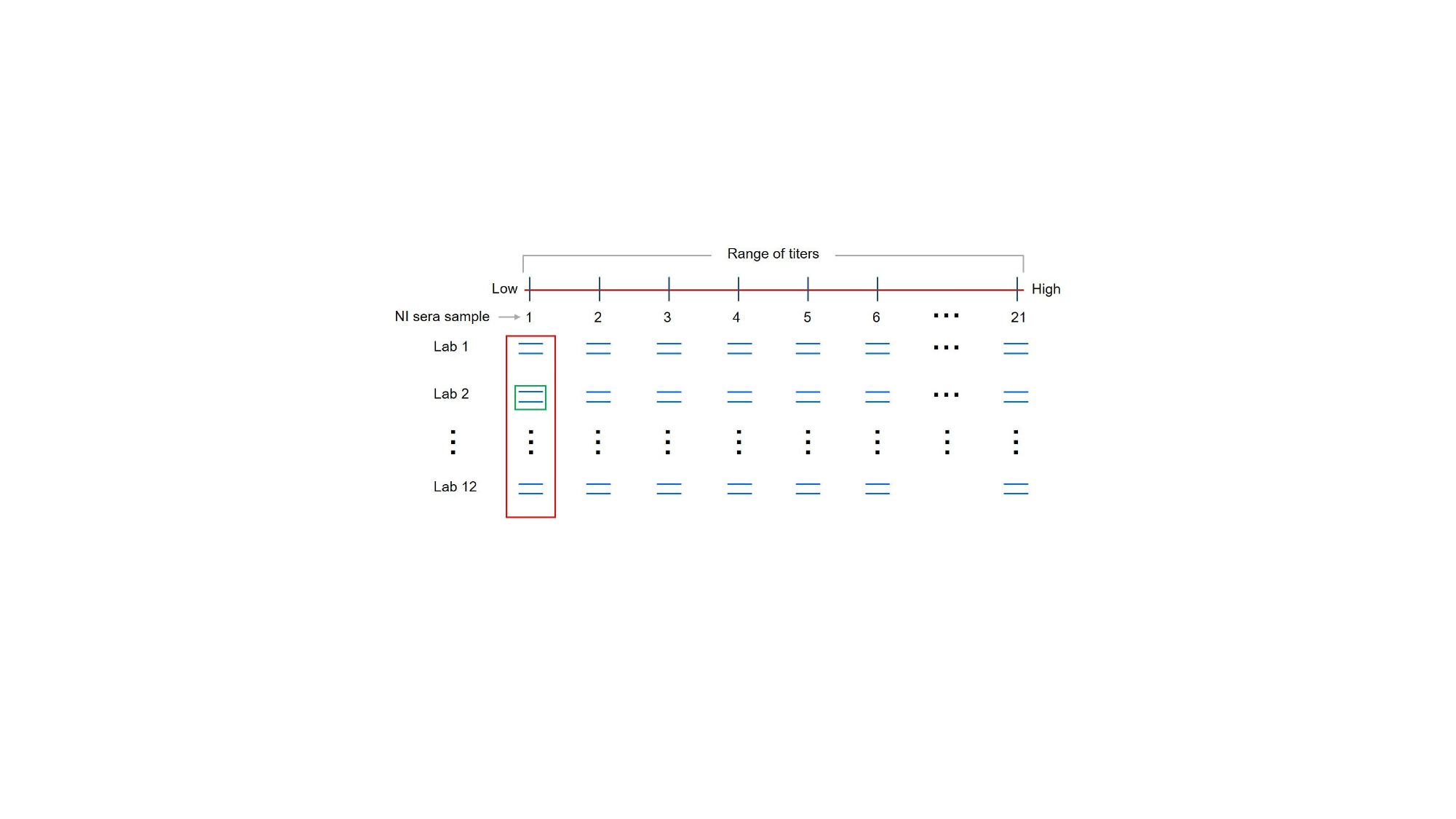

Supplement: Supplementary Fig. 2 — Diagram illustrating how within-sample coefficient of variation values were calculated. Each laboratory (Lab 1 to 12) provided titer data for each of the 21 duplicated NI sera samples in the specimen panel. Each blue bar in the diagram represents a titer result for an NI sera sample replicate. As an example, we first calculated within-sample CV values which measured the deviation of each individual titer value for NI sera sample 1 assayed by Lab 2 from the mean of the two titer values for that same sample (green box). This identical method was used to calculate within-sample CV values for each of the other labs. These values were then pooled over all 12 laboratories within that sample (red box) to calculate an overall within-sample CV value for the laboratories for NI sera sample 1 using a mixed model analysis of variance (ANOVA) with repeated measures. Sample, lab, and their interaction were modeled as random effects in this analysis. This identical method was used to calculate the overall within-sample CV variance values for the laboratories for each of the other NI sera samples. See Fig. 2b for results of this analysis. [file mmc4.pptx]

## Slide 1
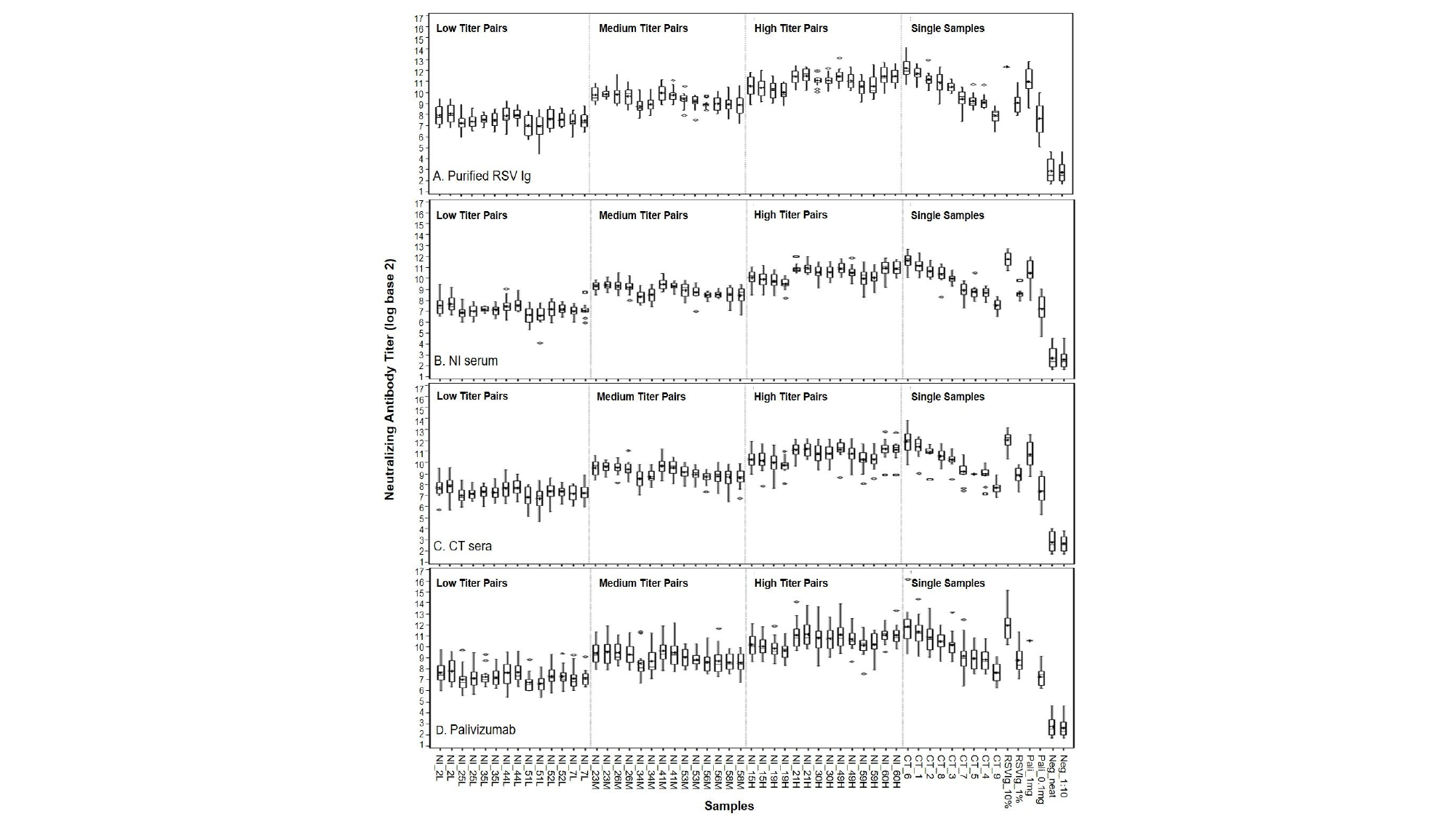

Supplement: Supplementary Fig. 3 — Box plots illustrating degree of agreement after normalization. The box plots display the degree of agreement on RSV neutralizing antibody (nAb) titer for each sample after normalization using A) purified RSV immune globulin (Ig) (sample RSVIg_10%); B) natural infection (NI) serum (sample NI_56M); C) clinical trial (CT) sera (sample CT_5); and D) palivizumab (sample Pali_1mg) as the pseudo internal standard. In these plots, the box is defined by the interquartile range (IQR)—the 25th and 75th percentiles of the distribution; the horizontal line within the box represents the median (50th percentile); and the asterisk represents the mean. Vertical lines extend to the most extreme observation that is less than 1.5×IQR, and the open diamonds correspond to moderate outliers (between 1.5-3.0×IQR). The 21 replicated NI serum samples are divided into low, medium, and high nAb titer groups of 7 samples per group on the left-hand side of each plot. The 15 single (non-replicated) samples are grouped on the right hand side of each plot. [file mmc5.pptx]

## Slide 1
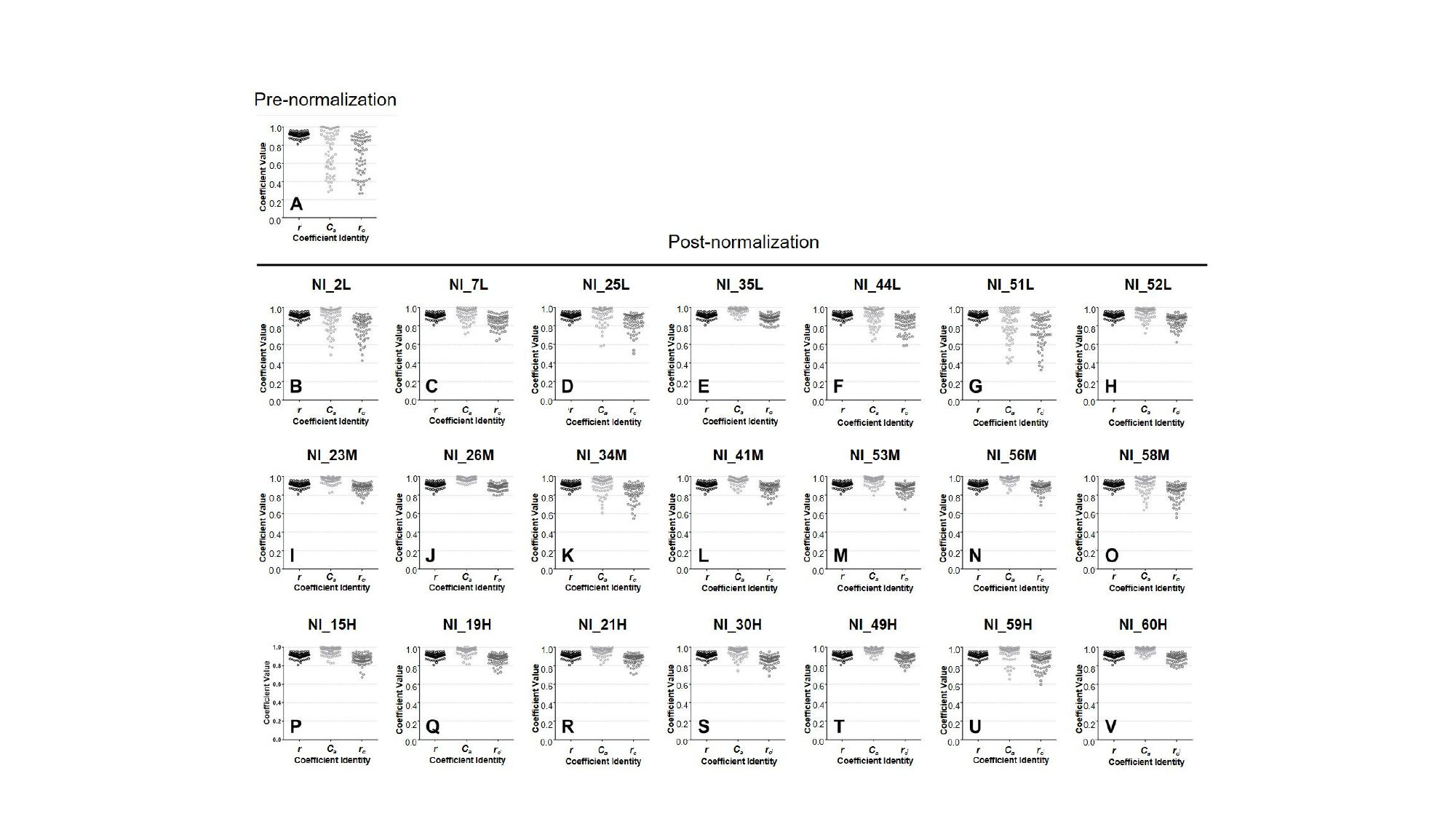

Supplement: Supplementary Fig. 4 — Graphs of statistical coefficient values pre- and post-normalization using each natural infection sera sample as a pseudo internal standard. In each graph, the values of the statistical coefficients used to quantify precision (r), agreement (Ca), and overall agreement (rc,) are plotted in aggregate. Each open circle plots the value of the indicated statistical coefficient (r, Ca, or rc) for the comparison of antibody titer results for all samples from a pair of laboratories. The values of these statistical coefficients prior to harmonization are plotted in graph A. The values of these statistical coefficients after normalization using each of the 21 natural infection (NI) serum samples (2L, 7L, 15H, 21H, 23M, 25L, 26M, 30H, 34M, 35L, 41M, 44L, 49H, 51L, 52L, 53M, 56M, 58M, 19H, 59H, and 60H) as a pseudo internal standard for normalization are plotted in graphs B through V. The post-normalization graphs are grouped by the RSV neutralization titer of the NI sera samples used for normalization as follows: low titer (graphs B to H); medium titer (graphs I to O); and high titer (graphs P to V). [file mmc6.pptx]
